# Proposed grading scheme for inflammatory bowel disease in ferrets and correlation with clinical signs

**DOI:** 10.1177/1040638719896555

**Published:** 2020-01

**Authors:** Paola Cazzini, Megan K. Watson, Nicole Gottdenker, Joerg Mayer, Drury Reavill, James G. Fox, Nicola Parry, Kaori Sakamoto

**Affiliations:** Departments of Pathology (Cazzini, Gottdenker, Sakamoto), College of Veterinary Medicine, University of Georgia, Athens, GA; Small Animal Medicine and Surgery (Mayer), College of Veterinary Medicine, University of Georgia, Athens, GA; Department of Animal Health and Conservation, Zoo New England, Boston, MA (Watson); Zoo/Exotic Pathology Service, Carmichael, CA (Reavill); Division of Comparative Medicine, Massachusetts Institute of Technology, Cambridge, MA (Fox, Parry); Current address: Easter Bush Pathology, Royal (Dick) School of Veterinary Studies, The University of Edinburgh, Edinburgh, UK (Cazzini)

**Keywords:** clinical signs, ferrets, grading scheme, histopathology, inflammatory bowel disease, *Mustela putorius furo*

## Abstract

Inflammatory bowel disease (IBD) is an idiopathic, chronic, inflammatory disease of the gastrointestinal tract of companion animals, including ferrets (*Mustela putorius furo*). Clinical signs of IBD are nonspecific, and intestinal biopsies are necessary for a definitive diagnosis. A grading scheme has not been established for ferrets. Additionally, the association between histologic severity and clinical signs in ferrets is unknown. We evaluated enteric samples from ferrets diagnosed with IBD, compared histologic grading schemes, and correlated the results with the severity of clinical signs. Enteric sections from 23 ferrets with IBD were analyzed using grading schemes for intestinal inflammation in cats and dogs, and a correlation with clinical signs was evaluated. After dividing the histologic samples into groups based on the severity of clinical signs, main histologic differences were identified. Age and sex were also assessed for correlation with clinical signs. No significant correlation was found between the 2 grading schemes and clinical signs (rho = 0.02, *p* = 0.89; rho = 0.26, *p* = 0.18, respectively). Degree of villus fusion, hemorrhage and/or fibrin, epithelial damage, inflammation density, and crypt abscess formation were used retrospectively to create a ferret IBD grading scheme, which was significantly correlated with the severity of clinical signs (rho = 0.48, *p* = 0.01). A positive correlation was observed between age (*p* = 0.04) and females (*p* = 0.007) with severity of clinical signs. Our ferret grading scheme may have clinical utility in providing a more objective, consistent evaluation of IBD in ferrets.

## Introduction

Inflammatory bowel disease (IBD) is an idiopathic, chronic, inflammatory disease of the gastrointestinal tract affecting multiple species, including cats, dogs, and humans.^6,18,28^ Although virtually unheard of a decade ago, IBD is now considered the most common, yet poorly described, disease in ferrets (*Mustela putorius furo*).^4^ IBD is usually diagnosed in ferrets > 1 y old, and many ferrets are asymptomatic or demonstrate only subtle signs of illness that are often overlooked by the owners.^4,5,16^ Clinical signs of IBD are nausea (manifested by bruxism and drooling); anorexia; chronic weight loss; green-to-brown, mucoid-to-“birdseed” (“seeds” represent undigested globules of fat and proteins) diarrhea; melena; proctitis; and rectal prolapse.^4,16^ If untreated, IBD can progress to lymphoma.^4,5^ The distinction between IBD and lymphoma necessitates histologic analysis, and, in more severe cases of IBD, it may also require the aid of immunohistochemistry.^29^

The etiology of IBD in ferrets, as in other species, is unknown. It is hypothesized that the underlying cause involves a complex interaction between microorganisms, dietary proteins that are not part of the diet in the wild for this species, and the mucosal immune system.^4^ This interaction in susceptible patients results in a chronic inflammatory response. Known causes of gastritis and/or enteritis in ferrets include: *Helicobacter mustelae*, ferret enteric coronavirus, *Lawsonia intracellularis* (proliferative ileitis and/or colitis), cryptosporidiosis, coccidiosis, and foreign body ingestion.^5,10–12,24^ These pathogens or intestinal microbiota may trigger an aberrant immune response that may continue long after the primary cause has resolved. A thorough clinical history may aid in the diagnosis of IBD. Information regarding contact with new ferrets may suggest the introduction of a new pathogen, and information regarding the duration of clinical signs, stool abnormalities, and decline in body condition may help in distinguishing other diseases from IBD, which is a gradual illness.^5^

In humans^33^ and dogs,^22,27^ defects in immune receptors for recognition of commensal bacteria resulting in an upregulation of inflammatory cytokines play a role in IBD susceptibility. However, it is unknown if genetic defects contribute to the development of IBD in ferrets and cats.^21^ Dietary factors may also play a role in the susceptibility of domestic dogs, cats, and ferrets to IBD.^4,19^ Ferrets are obligate carnivores and require a meat diet high in quality proteins and fat, and low in carbohydrates and fiber.^25^ It is possible that domestic ferret diets, including commercial formulations, may expose ferrets to novel proteins and changes in dietary components (e.g., protein, fat, carbohydrates) potentially triggering IBD. Although the exact cause of IBD is unknown, treatment for IBD in ferrets consists of the removal of the possible contributing factors, suppression of the inflammatory response with azathioprine or corticosteroids, and if present, resolution of secondary lesions such as ulcers, bacterial overgrowth, and chronic lymphocytic portal hepatitis or cholestasis.^4,5^

Microscopic examination of intestinal biopsies, ideally full-thickness, is necessary for definitive diagnosis of IBD.^4,6,9,16^ Although endoscopy is a less invasive procedure that allows visualization of the mucosal surface, it does not allow inspection of the mesenteric lymph nodes,^5^ or often the submucosa or muscular tunics, which may aid in the early detection of lymphoma, a possible sequela to IBD. Histopathologically, IBD is characterized by shrinkage and blunting of the intestinal villi and by an inflammatory infiltrate of the mucosa. The inflammatory cells present are predominantly lymphocytes and plasma cells, but less commonly, an eosinophilic component can be present.^4^ Inflammatory bowel disease with eosinophilic infiltrates should be distinguished from the less common, eosinophilic granulomatous disease that involves multiple organs and is not limited to the gut.^4,5,13,16^

Ideally, protocols and dosages for long-term treatment should be based on clinical signs and intestinal histopathology,^4^ but a universal grading scheme to analyze ferret IBD has not been established, creating confusion with regards to what should be considered mild, moderate, or severe IBD. Underestimation of IBD severity could lead to ineffective treatment protocols and possible progression to intestinal lymphoma, whereas overestimation could lead to unnecessary, and potentially life-threatening, drug side effects (such as immune or bone marrow suppression). A grading scheme is, therefore, necessary for more consistent and accurate disease assessment, and consequently, a more effective therapeutic plan.

Histologic interpretation in all species is subjective and suffers from inter-observer variability.^31^ For this reason, several grading schemes have been proposed for the evaluation of intestinal specimens of dogs and cats with intestinal inflammation.^1,8,19,23^ A universal grading scheme will make diagnoses more uniform among pathologists, granting more consistency. In addition, correlation between the degree of histologic lesions and severity of clinical signs would be most useful in assessing and managing patients. The association between the severity of the inflammatory infiltrate and clinical signs in ferrets is not known. Accordingly, we assessed in ferrets 2 grading schemes used in small animals for intestinal inflammation,^9,23^ and tested their correlation with clinical presentation. Additionally, we separated the intestinal samples based on the severity of clinical signs and designed a third grading scheme to more accurately reflect the correlation of clinical signs with histologic lesions in ferrets. Correlation of age and sex with severity of clinical signs was also tested.

## Materials and methods

### Study population

By searching the databases at Zoo/Exotic Pathology Service (Carmichael, CA) and Division of Comparative Medicine, Massachusetts Institute of Technology (Cambridge, MA), we identified 61 cases in which intestinal biopsy samples had been taken in ferrets and a diagnosis of IBD had been reached. The initial diagnosis of IBD was made by a veterinary pathologist with expertise in ferret pathology using criteria based on a 2006 study in cats.^9^ Briefly, IBD was diagnosed when various degrees of mucosal and submucosal infiltration by inflammatory cells (mainly small lymphocytes and plasma cells) were observed. Each case was accompanied by a brief clinical history. For all cases, an etiology for the intestinal inflammation was not identified. Often, multiple intestinal sections were available from the same ferret; however, because the small intestine was more consistently available, we elected to limit our study to this part of the intestinal tract. If multiple small intestinal sections were available, the one exhibiting the most severe histologic changes was used. Intestinal sections from 3 healthy ferrets with no clinical signs were used as controls. Inclusion criteria for our study included:

- Ferrets > 1 y old were included; IBD is rarely present in ferrets < 1 y old.^4^
- No other relevant disease identified in the history was present; all cases in which a secondary disease could have been the cause of all, or part, of the clinical signs described were excluded.
- Clinical history was available.
- Sample was full thickness and in adequate condition; if the histologic section was too small or contained large artifacts, it was excluded.

Among the initial 61 cases, 22 were excluded because of the presence of a severe secondary disease. Secondary diseases included lymphoma (10 cases), insulinoma (8 cases), intestinal carcinoma (2 cases), adrenal carcinoma (1 case), and suppurative pyelonephritis (1 case). Nine cases were excluded because the animals were < 1 y old or the age was not specified. In 3 cases, no clinical history was provided, and for 1 case, the sample was inadequate.

After applying the exclusion criteria, 23 cases of IBD and 3 control samples were included in our retrospective study. Twenty-one of the IBD specimens had been submitted to the Zoo/Exotic Pathology Service and 2 had been submitted to the Division of Comparative Medicine, Massachusetts Institute of Technology. Among the 23 ferrets, 6 were females and 17 were males. Age ranged from 1.5–6 y (mean 3; median 3.5). In the initial diagnosis, inflammation was considered severe in 8 cases, moderate-to-severe in 3 cases, moderate in 9 cases, and mild in 3 cases. Clinical history was available for all of the cases evaluated and included a variety of clinical signs ranging from weight loss, anorexia, and diarrhea, to sudden death.

### Slide preparation

Paraffin-embedded tissues were sectioned at 4–5 µm thickness, stained with routine hematoxylin and eosin (H&E), and examined microscopically by a board-certified, veterinary pathologist (K Sakamoto) initially blinded to the original diagnosis.

### Case evaluation

Based on the original recorded diagnosis, numerical values were assigned to the severity of the inflammation observed (severe = 3; moderate-to-severe = 2.5; moderate = 2; mild = 1). For the first grading scheme, the following parameters were recorded for all H&E sections, as in the 2011 study in cats^23^: location, distribution, and density of the lymphocytic population but with modifications as described below. Parameters used to differentiate inflammation from neoplasia, such as immunohistochemistry, had been used in a previous study^29^ to identify cases of lymphoma but were considered irrelevant for our study. The deepest location of infiltrates (i.e., which layer of the intestine farthest from the lumen was affected) was recorded as mucosa, submucosa, tunica muscularis, or serosa in each section. The density of mucosal lymphoid infiltrates, as determined by the number of lymphoid cells across the width of the villus lamina propria in the worst-affected villi, were classified as diffuse or multifocal. Diffuse infiltrates were further divided into low, medium, or high density. Lymphocytic infiltrates within the epithelium were classified as being present in the surface (villi), in the crypts, or in both locations. Intraepithelial infiltrates were further categorized based on their distribution as single cells, nests, or plaques. In addition, the lymphocytic infiltrates were evaluated for monomorphism versus polymorphism.^23^ Descriptive terms were converted into numerical values attributing higher values to more severe manifestations of inflammation (Table 1).

**Table 1.** Parameters modified from the first grading scheme in a 2011 study^23^ for cats and used to score the intestinal lesions in ferrets with inflammatory bowel disease.

Score	0	1	2	3	4
Location	Absent	Mucosal	Submucosal	Tunica muscularis	Serosal
Density of mucosal infiltrates					
If diffuse	Absent	Low density (< 2 cells)	Medium density (3–6 cells)	High density (> 7 cells)	
If multifocal	Absent	Present			
Infiltrates of T cells within epithelium	Absent	Surface	Crypts	Both	
Intraepithelial infiltrates	Absent	Single cells	Nests (≥ 5 clustered intraepithelial lymphocytes)	Plaques (≥ 5 adjacent epithelial cells overrun by lymphocytes)	
Lymphocyte morphology	Monomorphic	Polymorphic			

For the second grading scheme, the following parameters were recorded for all H&E sections, according to the 2008 study in dogs and cats^8^ and modified in 2018 for dogs^1^ [originally following recommendations discussed by Willard and Mansell in 2012 (Willard M, Mansell J. Thoughts behind the WSAVA GI grading system. Proc 63rd Ann Meeting Am College Vet Pathol and 47th Ann Meeting Am Soc Vet Clin Pathol; Dec 2012; Seattle, WA)], villus stunting, crypt abscesses, and lymphangiectasia (Table 2).

**Table 2.** Parameters modified from the second grading scheme in a 2018 study^1^ (originally adapted from Willard M, Mansell J. Thoughts behind the WSAVA GI grading system. Proc 63rd Ann Meeting Am Coll Vet Pathol and 47th Ann Meeting Am Soc Vet Clin Pathol; Dec 2012; Seattle, WA) for dogs and used to score the intestinal lesions in ferrets with inflammatory bowel disease.

Score	0 = absent	1 = mild	2 = moderate	3 = severe
Villus blunting	No villus blunting	Villi reduced to 75% of normal length	Villi reduced to 50% of normal length	Villi reduced to 25% of normal length
Crypt distention	No crypt distention	10% of crypts distended	25% of crypts	50% of crypts
Lymphangiectasia	No lacteal dilation	Lacteal ≤ 50% of width of the villus	Lacteal ≤ 75% of width of the villus	Lacteal ≤ 100% of width of the villus

A degree of clinical severity was assigned to each case based on the clinical signs reported (Table 3). Inappetence, mild weight loss or dehydration, and occasional vomiting were considered mild clinical signs; chronic weight loss, moderate dehydration or diarrhea, and frequent vomiting were considered moderate clinical signs; severe weight loss and diarrhea, emaciation, critical illness, or death, were considered severe clinical signs. Sex and age were correlated with the severity of clinical signs.

**Table 3.** Grading scale of clinical signs of ferrets with inflammatory bowel disease based on severity.

Degree	Absent (0)	Mild (1)	Moderate (2)	Severe (3)
Clinical signs described	No GI-related clinical signs	• Mild weight loss • Inappetence • Mild dehydration • “Not doing well” • Occasional vomiting	• Chronic weight loss • Moderate diarrhea • Moderate dehydration • Frequent vomiting	• Severe weight loss and diarrhea • Critically ill • Found dead • Emaciated

For our retrospectively applied grading scheme for ferrets, samples were divided into groups based on the degree of clinical signs. The slides were examined in these groups based on clinical signs, and new criteria that appeared subjectively different between the groups were selected. After reviewing the slides by group, there were a few lesions that appeared to characterize each group. The degree of villus fusion (as determined indirectly by crypt-to-villus number ratio), hemorrhage and/or fibrin, and epithelial damage (scored as in Table 4) were considered subjectively different between the groups, being less prominent in the group that had absent and mild clinical signs, and more prominent in the group that had severe clinical signs. Crypt-to-villus number ratio was used instead of villus blunting or stunting given that the latter 2 require a subjective comparison to “normal” villus length, whereas a crypt-to-villus number ratio can be objectively calculated within the sample itself. In addition, among the criteria used in the other 2 grading schemes, inflammation density (from the grading scheme by the 2011 study^23^) and crypt abscesses (modified from the grading scheme by the 2018 study^1^) appeared to differ with the severity of clinical signs. Given that crypt abscesses were relatively rare in the ferret samples, scoring was modified to reflect the severity of the lesion instead of the number (see Table 4). The slides were rescored once more in a blinded fashion using these parameters (Table 4; Supplementary Table 1).

**Table 4.** Parameters used in our retrospective grading scheme for inflammatory bowel disease in ferrets.

Score	0	1	2	3	4
Crypt-to-villus no. ratio	1:1	2:1	3:1	4:1	5+:1
Hemorrhage/fibrin	Absent	Focal	Multifocal	Locally extensive	Diffuse
Mucosal epithelial damage	Absent	Vacuolation/attenuation	Focal necrosis	Ulceration	
Inflammation density	Absent	Low density	Medium density	High density	
Crypt distention*	No crypt distention	Mild crypt distention (up to width of adjacent villus)	Moderate crypt distention (greater villus width, fluid accumulation)	Marked crypt distention with cell accumulation	

* Crypt abscesses were modified from a 2018 study^1^ to reflect the relative paucity of crypt abscesses in ferret lesions.

### Statistical analysis

The severity of inflammation present in the original histologic diagnosis was correlated with the degree of clinical signs using Spearman rank correlation. The descriptive data were converted into numerical values and summed, so that higher severity corresponded to higher total score (Tables 1–3). Each scoring scheme total and each element of the scoring scheme totals (Supplementary Table 1) were tested for normality by a Shapiro–Wilk test. Because all scores, with the exception of the total for the third scoring scheme, were non-normally distributed, Spearman rank correlation tests were used to evaluate associations between each element of each scoring scheme (2011 study,^23^ 2018 study,^1^ and our retrospective grading scheme) and the representative totals for each of these scoring schemes and the clinical sign severity score.

The final scores on the retrospective grading scheme based on 3 new criteria (crypt-to-villus number ratio, hemorrhage or fibrin, and epithelial damage), and on 2 criteria from the previously tested grading scheme (inflammation density, from the 2011 study in cats,^23^ and crypt abscesses, modified from the 2018 study in dogs^1^) were added and then correlated to the severity of clinical signs. Spearman rank correlation was used to determine the correlation between the score obtained with our ferret grading scheme and the severity of clinical signs.

The severity of inflammation present in the original histologic diagnosis was correlated with the final scores on our ferret grading scheme using Spearman rank correlation. The correlation between severity of clinical signs with age and sex of the ferrets was assessed using Spearman rank correlation and Wilcoxon rank sum test with continuity correction, respectively. The statistical software R v.3.2.2 (https://www.r-project.org/) was used for all analyses.

## Results

In 2 cases, clinical signs were absent. Three cases were mild, 15 were moderate, and 3 cases had severe clinical signs. The assessment of the severity of inflammation present in the original histologic diagnosis was not significantly correlated to the severity of clinical signs (rho = 0.24, *p* = 0.27, Spearman rank correlation).

**Figure 1.**
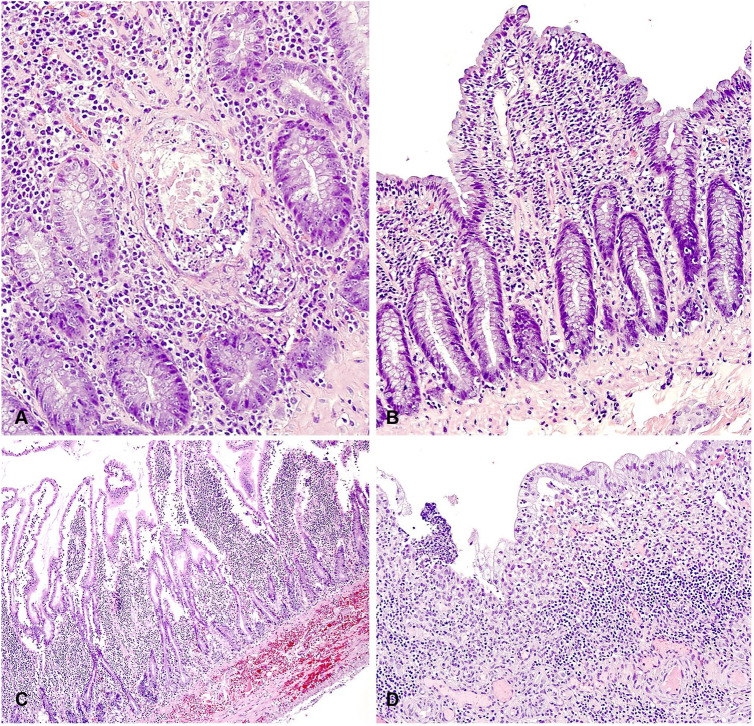
Representative photomicrographs of lesions in the small intestine of ferrets with inflammatory bowel disease. These features highlight components of our retrospective grading scheme: **A.** Crypt abscess, representative of a score of 3. **B.** Increased crypt-to-villus number ratio, score of 4. **C.** Hemorrhage, score of 3. **D.** Epithelial damage, score of 3. H&E.

Neither of the first 2 grading schemes was significantly associated with clinical severity. The correlation between the severity of clinical signs and the grading scheme for intestinal inflammation according to the 2011 study^23^ was performed with Spearman rank correlation (rho = 0.02, *p* = 0.89). The correlation between the severity of clinical signs and 3 criteria (villus stunting, crypt abscesses, and lymphatic dilation) used to assess intestinal inflammation in the 2018 study^1^ was also performed with Spearman rank correlation (rho = 0.26, *p* = 0.18). Each individual parameter from the first 2 schemes was then assessed to determine if they could be useful in the development of our ferret grading scheme. Of these, only crypt abscesses (modified from the 2018 study^1^; rho = 0.46, *p* = 0.02) were significantly correlated with the clinical severity score. Although inflammation density was not correlated with the clinical outcome (rho = 0.33, *p* = 0.12), we felt this parameter to be biologically relevant and critical to the original diagnosis of the disease. These 2 parameters were therefore used to provide additional histologic parameters for the retrospective scheme. Lesions representing part of the retrospective grading scheme are crypt abscess (Fig. 1A, score of 3), villus-to-crypt number ratio (Fig. 1B, score of 4), hemorrhage (Fig. 1C, score of 3), and epithelial damage (Fig. 1D, score of 3).

There was a significant, positive correlation between our retrospective grading scheme and clinical severity score (Spearman rank correlation, rho = 0.48, *p* = 0.01). The assessment of the inflammation severity present in the original histologic diagnosis (severe, moderate-to-severe, moderate, and mild) was also significantly correlated with the final scores of our ferret grading scheme (Spearman rank correlation, rho = 0.60, *p* = 0.002).

There was a positive correlation between ferret age and IBD clinical severity score (Spearman rank correlation, rho = 0.44, *p* = 0.04). There was a significant difference between sex (male vs. female, not accounting for spayed or neutered) and IBD clinical severity score, with females having a higher IBD clinical severity score than males (Wilcoxon rank sum test with continuity correction, w = 84, *p* = 0.007).

## Discussion

No correlation was observed between either of the 2 histologic grading schemes evaluated (both developed for small animals)^1,23^ and the severity of clinical signs reported. This may be the result of several reasons. In veterinary medicine, the recorded clinical signs heavily rely on what is observed by the owners. There is great variability in the owner’s attention to changes in pet behavior and for manifestations of pain, resulting in a potentially inaccurate and disparate collection of clinical signs in our study. Additionally, recognizing discomfort or pain in a ferret is often difficult^4^ and requires careful observation of an undisturbed animal.^26^ A list of specific questions could be given to the owners in a prospective study to minimize these differences. Correlating histologic lesion severity and clinical severity is important for the clinician, however, as this will better inform their treatment plan. Additionally, these grading schemes had been developed for cats^23^ and dogs,^1^ and may not be useful for ferrets.

Crypt distortion, villus blunting and fusion, and fibrosis were most commonly seen in cats with moderate or severe IBD in one study,^2^ and abnormalities in mucosal architecture, principally villus fusion and atrophy, were correlated with the score for the clinical signs in another study on IBD in cats.^17^ Correlation between clinical signs assessed using a canine IBD activity index (CIBDAI) and histopathologic changes was successful in a 2003 study.^20^ However, controversy still exists in the correlation between histology and clinical signs of IBD.^30^ Part of the problem is the result of inter-observer variation in histologic evaluations of intestinal tissues.^31^ Additionally, the absence of a commonly accepted grading scheme for small animal enteric lesions has created disparity in diagnosis. Attempts have been made to promote universal criteria for histologic evaluation of enteric samples^8^; however, even with this grading scheme, differences in tissue processing (such as staining intensity and coloration) still create disagreement among pathologists, especially in distinguishing mild and moderate processes.^32^ Collection of surgical, rather than endoscopic, biopsy samples yields a more accurate diagnosis in enteric samples from cats and dogs and is preferable.^6,9^ In our study, we included only full-thickness enteric biopsies.

Another limitation in correlating clinical signs with histopathologic findings may be a result of the failure to biopsy the relevant area of the intestine that is causing the clinical signs.^7^ In our study, when multiple samples were available from the same animal, the one exhibiting the most severe histologic changes was examined. Unfortunately, in some cases, only one sample of intestine was available for evaluation, and the possibility of having missed a more severe lesion or representative section exists. This approach may have introduced additional bias into our study; however, we wanted to ensure that we had the richest availability of lesions to examine. Random choice of sampling may have been less biased but would have increased our likelihood of missing a lesion that would have correlated with clinical severity. Our study was also limited in that samples were not taken from the same section of intestine, and the exact location of the sampling was not provided.

Given the retrospective nature of our ferret grading scheme, a positive correlation with clinical signs was expected, although the pathologist using the scheme was again blinded to the original diagnosis of each case. Regardless, the identification of histologic characteristics that correlate with the severity of clinical signs is significant. Although hemorrhage and fibrin are not typically histopathologic features of IBD, these lesions were observed more often in clinically severe cases. It is possible that these lesions reflect a separate process; however, completely ruling out other diagnoses was beyond the scope of our study. The specific location of the hemorrhage and/or fibrin was also not specifically differentiated, given that only a few cases exhibited this lesion. A prospective study will be needed to test the validity of our grading scheme and its true correlation with clinical severity.

There was no significant correlation between the original grading scheme, performed by pathologists with ferret expertise, and the clinical severity score (rho = 0.24, *p* = 0.27), but our ferret grading scheme did show a significant correlation with clinical severity score (rho = 0.48, *p* = 0.01). This is likely because our ferret scheme combines an objective and quantifiable set of histopathologic criteria to generate a grading score, which was specifically designed to better reflect clinical severity. It is our hope that providing a grading scheme will be useful to pathologists who do not examine ferret tissues on a regular basis and will allow for inter-pathologist comparisons.

In our study, a positive correlation between clinical sign severity score for IBD and ferret age was observed. This may be the result of the prolonged effects of this chronic disease accumulating over time or to a decrease in pain tolerance in older animals. Increase in age was observed to decrease pain perception in a human study.^15^ In a study on 58 dogs and 26 cats with IBD, no age predisposition was observed^19^; however, severity of clinical signs did not correlate with the age of the animal. It is also possible that aging animals may be affected by concurrent diseases contributing to their clinical signs, although we tried to rule out this possibility when multiple tissues or diagnoses were provided.

In our study, female ferrets had a higher clinical score for IBD than males. Although the literature is often controversial regarding the effects of sex over sensitivity to pain in humans, females are generally considered more sensitive to noxious stimuli and are at higher risk for many common pain conditions.^3^ In a 1992 study, no sex predisposition for IBD was observed in either dogs or cats,^19^ and sex was not reported to affect the density of immune cells in the lamina propria or epithelium in dogs with enteropathies.^14^

Limitations of our study include a small sample size available for analysis. Unfortunately, the frequent concomitant occurrence of other diseases in ferrets with IBD leads to exclusion of a high number of cases because of a possible confounding effect on clinical signs. Furthermore, because ours was a retrospective study, the clinical history was often limited and was not always centered on the gastrointestinal signs. The use of a questionnaire with guided questions may be advisable in prospective studies. Additionally, standardization of sampling sites and the examination of multiple biopsy samples from the intestine of the same patient may avoid missing the most severe lesion responsible for the clinical signs. The examination of only small intestinal samples is another limitation of our study. In a 2003 study^20^ on canine IBD, inflammation was present in the small intestine in most dogs (46 of 58), whereas in only a small portion of cases, the inflammation was only present in stomach or colon (5 and 7 of 58, respectively). Additionally, the small intestine is considered the segment of the gastrointestinal tract that is most affected by IBD in cats.^18^

## Supplemental Material

Supplemental_material – Supplemental material for Proposed grading scheme for inflammatory bowel disease in ferrets and correlation with clinical signs Click here for additional data file. Supplemental material, Supplemental_material for Proposed grading scheme for inflammatory bowel disease in ferrets and correlation with clinical signs by Paola Cazzini, Megan K. Watson, Nicole Gottdenker, Joerg Mayer, Drury Reavill, James G. Fox, Nicola Parry and Kaori Sakamoto in Journal of Veterinary Diagnostic Investigation
