# Supplementary material for: Proposed grading scheme for inflammatory bowel disease in ferrets and correlation with clinical signs
Source: J Vet Diagn Invest. 2020 Jan;32(1):17–24. doi: 10.1177/1040638719896555 (PMC7003233; doi:10.1177/1040638719896555)
Supplement: Supplemental_material – Supplemental material for Proposed grading scheme for inflammatory bowel disease in ferrets and correlation with clinical signs [file Supplemental_material.pdf]

Cazzini P, et al. Proposed grading scheme for inflammatory bowel disease in the ferret and correlation with clinical signs

Supplementary Table 1. Raw data from all inflammatory bowel disease study subjects, including scores from all 3 grading schemes and clinical sign score.

ID	Sex	Age (y)	Original diagnosis severity	First grading scheme from Kiupel et al.[2]								Second grading scheme by Allenspach et al.[1]				Third retrospective grading scheme (+ inflammation density and crypt abscess)				Rank of clinical signs
				Location	Distribution*	Multifocal	Density (for diffuse)	Mono vs. polymorphic	Intraepithelial infiltrates	Grouping	Total	Villus stunting	Crypt abscess†	Lymphatic dilation	Total	Crypt-to-villus ratio	Hemorrhage or fibrin	Epithelial damage	Total	
1	F	6	3	1	D	NA	3	0	1	2	7	3	1	0	4	4	3	2	13	3
2	M	1.5	2.5	2	D	NA	2	1	1	1	7	1	0	1	2	3	2	0	7	2
3	M	>1	2	1	D	NA	3	1	1	3	9	2	0	2	4	2	0	0	5	0
4	M	>1	1	1	D	NA	3	1	1	1	7	2	0	1	3	1	0	0	4	2
5	CM	2	3	1	D	NA	2	1	1	3	8	1	0	0	1	3	0	0	5	1
6	CM	5	3	1	D	NA	3	1	1	2	8	3	0	0	3	4	0	1	8	2
7	CM	5	3	4	D	NA	3	1	1	1	10	3	0	0	3	4	0	3	10	2
8	M	2	2.5	1	D	NA	2	1	1	3	8	2	0	0	2	4	0	1	7	2
9	M	3	2	2	D	NA	3	1	1	3	10	3	0	1	4	4	0	1	8	2
10	M	3	2	1	D	NA	3	1	1	3	9	3	0	2	5	4	0	0	7	0
11	CM	2.9	3	1	D	NA	2	1	3	1	8	2	0	0	2	2	0	0	4	2
12	M	4	2.5	2	D	NA	3	1	1	3	10	3	0	0	3	4	2	2	11	2

13	CM	3	3	2	D	NA	3	1	3	1	10	3	0	0	3	4	0	1	8	2
14	SF	2.3	2	1	D	NA	2	1	3	1	8	1	0	1	2	3	0	0	5	2
15	CM	4.4	2	2	D	NA	3	1	3	3	12	3	0	0	3	4	0	1	8	1
16	F	3	2	2	D	NA	3	1	3	3	12	2	0	0	2	3	0	0	6	2
17	F	6	3	1	D	NA	3	1	3	1	9	2	3	0	5	4	0	1	11	3
18	CM	2.4	1	1	M	1	NA	1	3	1	6	2	0	1	3	1	0	1	2	1
19	F	3.4	2	1	D	NA	2	1	3	1	8	1	0	1	2	2	0	0	4	2
20	M	6	3	1	D	NA	3	1	3	3	11	3	1	3	7	4	0	2	10	2
21	F	5	1	1	M	1	NA	1	3	1	7	2	0	0	2	3	2	0	5	3
22	M	1.5	2	1	D	NA	2	1	3	1	8	1	0	2	3	1	0	0	3	2
23	CM	3	2	1	D	NA	2	1	3	1	8	1	0	0	1	2	2	1	7	2
24	Control 1			1	D		1	1	3	1	7	0	0	0	0	1	0	0	2	0
25	Control 2			1	D		2	1	3	1	8	1	0	0	1	2	0	1	5	0
26	Control 3			1	D		1	1	3	1	7	1	0	0	1	1	0	0	2	0

CM = castrated male; F = female; M = male; NA = not applicable; SF = spayed female.

* D = diffuse; M = multifocal.

† Crypt abscess scoring was modified from Allenspach et al. for the third grading scheme only, as explained in Table 4.

References

1. Allenspach KA, et al. Correlating gastrointestinal histopathologic changes to clinical disease activity in dogs with idiopathic inflammatory bowel disease. Vet Pathol 2018:56:435–443.

2. Kiupel M, et al. Diagnostic algorithm to differentiate lymphoma from inflammation in feline small intestinal biopsy samples. Vet Pathol 2011;48:212–222.
